# Pirfenidone Prevents Heart Fibrosis during Chronic Chagas Disease Cardiomyopathy

**DOI:** 10.3390/ijms25137302

**Published:** 2024-07-03

**Authors:** Tatiana Araújo Silva, Diane Thomas, Jair L. Siqueira-Neto, Claudia Magalhaes Calvet

**Affiliations:** 1 Cellular Ultrastructure Laboratory, Oswaldo Cruz Institute (IOC), FIOCRUZ, Rio de Janeiro 21040-360, RJ, Brazil; tatibio86@gmail.com; 2 Center for Discovery and Innovation in Parasitic Diseases, Skaggs School of Pharmacy and Pharmaceutical Sciences, University of California San Diego, La Jolla, CA 92093, USA; d4thomas@health.ucsd.edu (D.T.); jairlage@health.ucsd.edu (J.L.S.-N.)

**Keywords:** heart fibrosis, Chagas disease, pirfenidone

## Abstract

Cardiac fibrosis is a severe outcome of Chagas disease (CD), caused by the protozoan *Trypanosoma cruzi*. Clinical evidence revealed a correlation between fibrosis levels with impaired cardiac performance in CD patients. Therefore, we sought to analyze the effect of inhibitors of TGF-β (pirfenidone), p38-MAPK (losmapimod) and c-Jun (SP600125) on the modulation of collagen deposition in cardiac fibroblasts (CF) and in vivo models of *T. cruzi* chronic infection. Sirius Red/Fast Green dye was used to quantify both collagen expression and total protein amount, assessing cytotoxicity. The compounds were also used to treat C57/Bl6 mice chronically infected with *T. cruzi*, Brazil strain. We identified an anti-fibrotic effect in vitro for pirfenidone (TGF-β inhibitor, IC50 114.3 μM), losmapimod (p38 inhibitor, IC50 17.6 μM) and SP600125 (c-Jun inhibitor, IC50 3.9 μM). This effect was independent of CF proliferation since these compounds do not affect *T. cruzi*-induced host cell multiplication as measured by BrdU incorporation. Assays of chronic infection of mice with *T. cruzi* have shown a reduction in heart collagen by pirfenidone. These results propose a novel approach to fibrosis therapy in CD, with the prospect of repurposing pirfenidone to prevent the onset of ECM accumulation in the hearts of the patients.

## 1. Introduction

Chagas disease, caused by the protozoan parasite *Trypanosoma cruzi*, affects 6 to 7 million people worldwide [1,2]. Traditionally, it is considered an endemic condition of Latin America. Nowadays, the disease is being referred to as an emerging infection because of migratory population flow, creating a new economic, political and social challenge in countries previously not classified as endemic to the disease [3]. Up to 110,000 cases of Chagas disease are estimated in Europe, with a high prevalence in Spain and Italy [4]. In the USA, alarming statistics of 300,000 infected patients and reports of autochthonous transmission through infected vectors found in southern states draw attention to this previously overlooked condition [5]. Chagas disease is one of the highest impact infectious diseases in the Americas, being an economic burden that reaches USD 7.19 billion a year, that emerges from lost productivity and premature mortality caused by its underlying cardiomyopathy. About 10% of these costs emanate from USA and Canada, indicating that Chagas disease has expanded to non-endemic areas [6,7].

*T. cruzi* infection can cause severe symptoms, leading to significant mortality in children during the acute phase and heart pathologies in chronic adults. Among Chagas patients, 5% to 10% present digestive involvement with risk of mega colon and mega esophagus and about 30% develop cardiac forms of Chagas disease [8]. Interstitial fibrosis seems to be a determinant factor for the pathogenic manifestations of Chagas disease. Clinical trials using cardiac magnetic resonance imaging with gadolinium enhancement, which allows visualization and measurement of fibrotic areas of the heart, showed a strong correlation between the percentage of tissue fibrosis and low ventricular ejection fraction index with a higher incidence of arrhythmias [9,10]. Recent observational clinical studies showed that the myocardial native T1 and extracellular volume values measured by cardiac MRI, which are indicators of heart fibrosis, were highly correlated with Chagas disease severity, being potential biomarkers of disease progression [11].

Currently, only two drugs are used against Chagas disease, benznidazole and nifurtimox. However, the use of these drugs requires long treatment, triggering severe adverse effects in patients, which often results in treatment suspension [12]. Benznidazole reduces the parasitic load during the chronic phase with variable efficacy, but when administered to patients with advanced cardiomyopathy, it did not result in an improvement in the clinical outcome [13]. A low proliferating form of *T. cruzi* was reported and might explain the clinical failure of compounds designed to targets involved in parasite proliferation. The detection of dormancy in *T. cruzi* parasites raises concerns about current methods for finding curative drugs and brings the need to develop alternative therapeutic approaches [14]. In parallel, the development of combination therapy has been suggested as a solution for Chagas disease treatment by multidisciplinary teams and has been identified as a major research priority for CD by the World Health Organization [15]. These facts, together with the implication of cardiac fibrosis to Chagas cardiomyopathy, suggest that the development of therapies aiming at fibrosis recovery are necessary in combination with trypanocidal agents to provide a significant clinical improvement when cardiomyopathy is advanced.

Several cytokines involved in fibrosis establishment have been described as exerting important roles in the development of Chagas disease pathology. Specifically, transforming growth factor β (TGF-β), tumor necrosis factor-α (TNF-α) and interferon gamma (IFN-γ) have also been proven to be key players in the immune response and pathogeny of Chagas disease [16,17,18]. Aiming to understand the regulation of extracellular matrix (ECM) during *Trypanosoma cruzi* infection, the previous work from our group showed that despite the increase in extracellular matrix observed in vivo, cardiomyocytes and cardiac fibroblasts highly infected with *T. cruzi* in vitro have low fibronectin expression, even after the exogenous addition of TGF-β and TNF-α to mimic in vivo inflammatory milieu [19,20,21]. The addition of TGF-β and TNF-α triggers an increase in the expression of extracellular matrix, specifically in non-infected cardiomyocytes of the infected culture [19]. There is no consensus on which cytokines are determinant for the development of cardiomyopathy and digestive outcomes, with different patients displaying high levels of different cytokines [22,23].

Signaling pathways triggered by TGF-β, TNF-α and IFN-γ may modulate the synthesis of ECM components underlying Chagas fibrosis. TGF-β triggers alternative signaling pathways to its classical pathway of SMADs [24], which may modulate the outcome of signaling and contribute to the remodeling of the ECM and development of fibrosis in Chagas disease. These alternative pathways include c-Jun N-terminal kinase (JNK1, JNK2, JNK3) and p-38MAPk [25]. Some authors suggest that the fate of TGF-β signaling, whether apoptosis, proliferation, differentiation or accumulation of ECM, may depend on which non-SMAD pathway is being stimulated together with the canonical pathway [25]. Data from our group showed that p-38MAPk and c-Jun pathways are activated by *T. cruzi* infection in cardiac fibroblasts, with higher levels of phosphorylation after TGF-β treatment, associated with increased fibronectin expression. These facts suggest that p38 and c-Jun regulate the fibrosis process mediated by TGF-β after *T. cruzi* infection [21]. Interestingly, these MAPK pathways are also activated by IFN-γ and TNF-α and may work together for the remodeling of extracellular matrix leading to Chagas fibrosis when different cytokines are being overexpressed. To investigate if the TGF-β signaling pathways, p-38MAPk and c-Jun can be targets for Chagas fibrosis treatment, we elected available small molecule inhibitors developed against these mediators, respectively, pirfenidone, losmapimod and SP600125. Pirfenidone and losmapimod are strong candidates for drug repositioning. 

Pirfenidone is an FDA-approved drug to treat pulmonary fibrosis. It also shows an anti-fibrotic effect in the heart and targets inflammatory signaling pathways, lowering TGF-β [26,27]. Pirfenidone could reduce collagen type 1 and MMP-2 stimulus by TGF-β in a 3D in vitro model of human cardiac fibrosis [28]. In rodent models of cardiomyopathy, pirfenidone reduced cardiac hypertrophy inhibiting activation of the JAK-2/STAT3 signaling pathway [29] and alleviated cardiac fibrosis induced by thoracic aorta constriction in mice, reducing collagen, α-smooth muscle actin and vimentin increase after the surgical procedure [30]. Left atrial fibrosis in a canine model of congestive heart failure was attenuated by pirfenidone treatment, which correlated with decreased levels of TGF-β and TNF-α, reduced activity of metalloproteinase-9 and increased TIMP-4 levels [31]. A recent study evaluated the efficacy of pirfenidone in patients with heart failure and preserved left ventricular ejection fraction, a condition in which myocardial fibrosis is associated with adverse outcomes [32]. Using magnetic resonance to measure cardiac extracellular volume (ECV), the trial showed that pirfenidone reduced myocardial fibrosis in patients after 52 weeks of treatment [32]. 

Losmapimod, a specific inhibitor of p38 MAPK, went up to phase 3 clinical trials for treatment of cardiomyopathies after myocardial infarction and currently is on Phase 1 to treat facioscapulohumeral dystrophy [33,34]. Early studies showed that losmapimod reduced cardiac hypertrophy and remodeling in spontaneously hypertensive stroke-prone rats [35]. In a Phase 1 clinical study, the compound was safe, well tolerated by healthy patients and achieved functional plasma concentrations after IV infusion [36]. The treatment of atherosclerosis patients with losmapimod resulted in reduced vascular inflammation and an overall systemic anti-inflammatory effect [37]. A double-blind, randomized, placebo-controlled Phase 2 trial with non-ST-segment elevation myocardial infarction patients treated with losmapimod revealed a reduction in high-sensitivity C-reactive protein and B-type natriuretic peptide [33]. However, in a Phase 3 trial, losmapimod was not capable of reducing the risk of major ischemic cardiovascular events when administered to patients with acute myocardial infarction [38], and clinical trials for losmapimod as a treatment for acute coronary syndrome were discontinued [39]. Because of its safety, tolerability and efficacy in reducing inflammation markers, losmapimod is now being evaluated as a treatment for facioscapulohumeral dystrophy [34].

SP600125 is a canonical JNK inhibitor well tolerated by mice and seems to provide protective effects on the heart in damaging conditions [40,41]. Treatment of HL-1 cardiomyocytes with SP600125 in models of hypoxia resulted in an attenuation of collagen 1A and 3A expression [42]. Dosing of mice with type 1 diabetes with SP600125 decreased inflammation mediators such as TNF-α and reduced cardiac fibrosis independently of hyperglycemia [43]. SP600125 is commonly used in research, never moved to clinical trials and was chosen as a proof-of-concept compound.

Our results show that all selected compounds could inhibit collagen stimulation by *T. cruzi* in cardiac fibroblasts, but only pirfenidone prevented the establishment of cardiac fibrosis in chronic models of Chagas cardiomyopathy.

## 2. Results

### 2.1. In Vitro Assays

Chagas disease fibrosis process is a multifactorial process triggered and sustained by inflammation, parasitic factors and persistence. In the chronic phase, once fibrosis is established, amastigote nests are not easily visualized and the amount of fibrosis is not proportional to infection. Therefore, we hypothesized that parasite antigens or their secreted factors could exert a paracrine effect, resulting in extracellular matrix modulation in cardiac fibroblasts in the development of cardiac fibrosis in Chagas disease. Trying to address all variables, we implemented assays using two approaches: (i) treatment of uninfected cardiac fibroblasts with parasitic factors (trypomastigote lysate or proteins/extracellular vesicles secreted by trypomatigotes) or with serum from infected mice in the chronic phase of infection; (ii) direct *T. cruzi* infection in immortalized human cardiac fibroblasts. We performed assays in multiwell plate format and used Sirius Red/Fast Green dye to quantify the collagen expression in cardiac fibroblasts by spectrophotometry.

**Figure 1:**
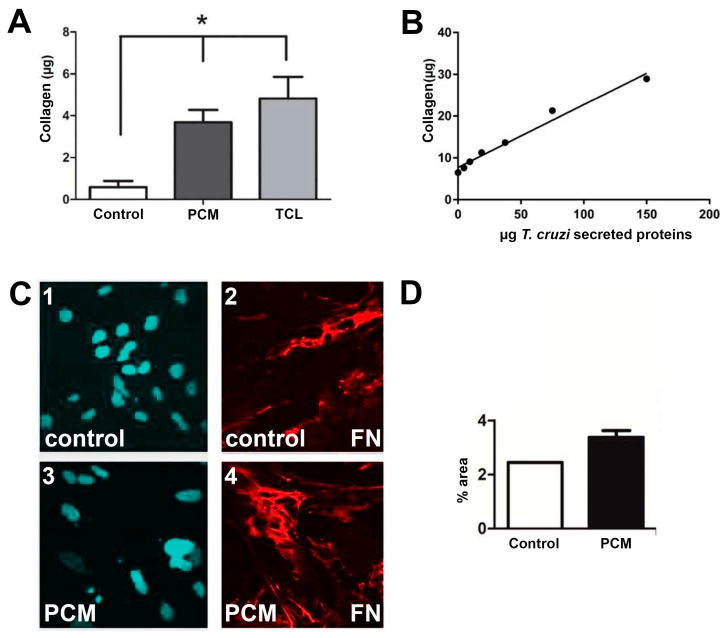
Proteins secreted by *T. cruzi* in the media (PCM—parasite-conditioned media) and *T. cruzi* lysate (TCL) stimulate collagen expression in human cardiac fibroblasts. (**A**) Analysis performed in 96-well format with collagen detection using Sirius Red/Fast Green shows that PCM and TCL treatment increases collagen in human cardiac fibroblasts; (**B**) the increase in collagen resulting from PCM stimulus is dose-dependent; (**C**) uninfected fibroblasts treated with PCM (C3–4) compared to untreated (C1–2) show the accumulation of fibronectin (FN) by immunofluorescence. DAPI is shown in blue and FN immunofluorescent staining in red; (**D**) image processing analysis showed an increase in the area occupied by fibronectin fibrils in the fields. * *p* ≤ 0.05 vs. control.

To evaluate the paracrine effect of *T. cruzi* factors on the expression of collagen and fibronectin in the extracellular matrix, human cardiac fibroblasts cultures were stimulated with 150 µg/mL of parasite-conditioned medium (PCM) or *T. cruzi* lysate (TCL). The treatment of cardiac fibroblasts with a single concentration of parasite-conditioned medium and *T. cruzi* lysate induced a rise in significant collagen in the extracellular matrix of uninfected human cardiac fibroblasts, detected with Sirius Red dye (Figure 1A). Interestingly, parasite-conditioned media (PCM, obtained after the overnight incubation of trypomastigotes in culture media, with posterior removal of parasites by centrifugation and filtration) lead to a dose-dependent stimulation in collagen expression in human cardiac fibroblasts, suggesting that parasites can modulate the extracellular matrix expression of adjacent non-infected cells in the tissue context independent of inflammation (Figure 1B). Fibronectin expression was also increased in human cardiac fibroblasts by incubation with parasite-conditioned medium, as shown by thicker deposits of this extracellular matrix protein visualized by immunofluorescent detection (Figure 1C) and the higher area of the images occupied by fibronectin staining after treatment with parasite-conditioned medium (Figure 1D).

**Figure 2:**
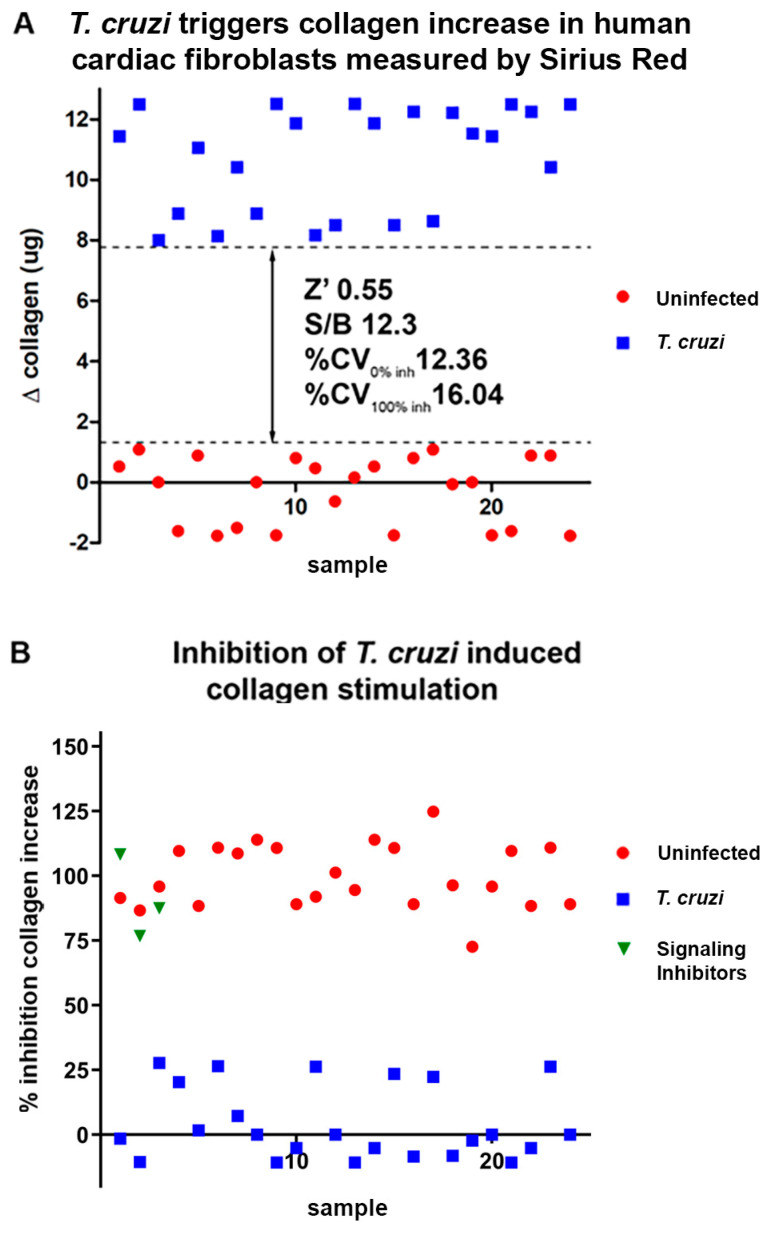
Scatter plots of data from Sirius Red/Fast Green assays for collagen detection directly infected with *T. cruzi*. (**A**) Raw data showing variation in collagen from uninfected controls to *T. cruzi*-infected samples. Δ collagen stands for *T. cruzi*-infected collagen values in µg minus uninfected control sample values (µg). *T. cruzi* infection robustly stimulates collagen in cardiac fibroblasts and resulted in Z’ = 0.55, S/B 12.3 and% CV 12.36–16.04. Z’- statistical parameter to assess reproducibility of high throughput assays [44]; S/B-signal to background ratio; % CV-percentage of mean by the coefficient of variation (i.e., the ratio of the standard deviation and the mean of the replicates); (**B**) normalized inhibition data showing that signaling inhibitors induced high inhibition of collagen stimulation by *T. cruzi* in human cardiac fibroblasts.

Raw data from assays infecting human cardiac fibroblasts directly with *T. cruzi* showed a robust and reproducible stimulus of collagen expression after *T. cruzi* infection. The statistical parameters to evaluate reproducibility of the assay reached a Z prime (Z’) higher than 0.5, signal to background ratio (S/B) of 12.3 and coefficient of variation (i.e., the ratio of the standard deviation and the mean of the replicates) at a maximum of 16.04% of the mean (Figure 2A). All values fell into the cut-off recommended by high-throughput screening guidelines, showing that the assay is robust and reproducible enough to be scaled up to high-throughput approaches [44]. Plotting the same data in normalized fashion displaying percent inhibition showed the same effect from a different perspective. Signaling inhibitors pirfenidone, losmapimod and SP600125 in a fixed dose also presented collagen inhibition higher than 75% (Figure 2B).

**Figure 3:**
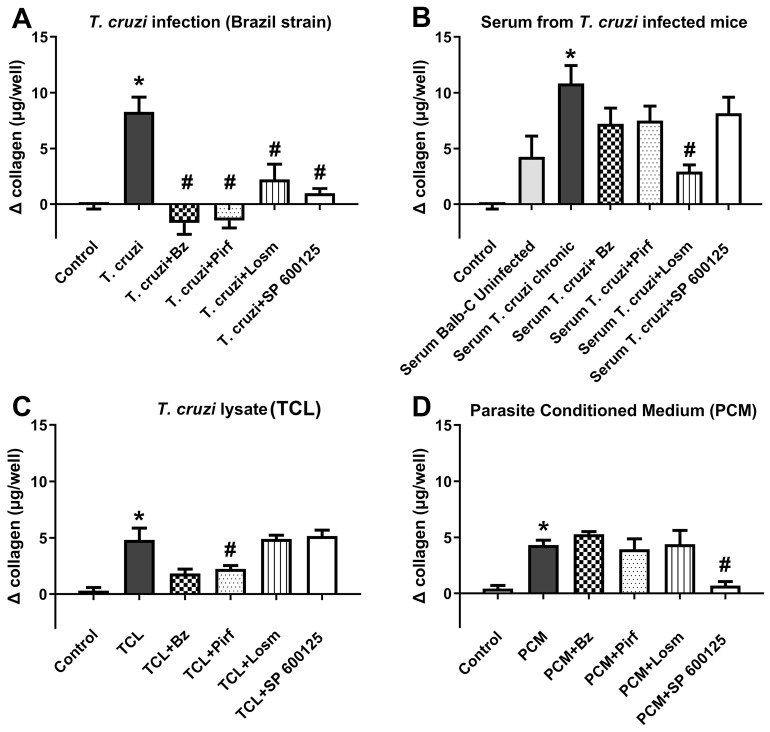
*T. cruzi* modulates collagen expression and inhibits the signaling inhibitors with collagen stimulation. (**A**) Infection with *T. cruzi* (Brazil strain), (**B**) stimulation with serum from uninfected and infected mice, (**C**) with the interaction of *T. cruzi* lysates and (**D**) with parasite-conditioned medium, resulted an increased collagen expression in human cardiac fibroblasts. Treatment with benznidazole, pirfenidone, losmapimod and SP600125 promoted a reduction in collagen expression in human cardiac fibroblast cultures infected by (**A**) *T. cruzi* and (**B**) stimulated with serum from uninfected and *T. cruzi*-infected mice. (**C**) The treatment with benznidazole and pirfenidone in human cardiac fibroblasts stimulated with parasite lysate promoted an inhibition of collagen expression in these cultures. (**D**) Human cardiac fibroblast cultures, treated with benznidazole, pirfenidone, losmapimod and SP600125, revealed that only the treatment with SP600125 inhibited the collagen expression in cells stimulated with parasite-conditioned medium. * *p* ≤ 0.05 vs. control; # *p* ≤ 0.05 vs. stimulus.

A significant rise in collagen in cardiac fibroblasts was detected in all conditions tested, i.e., *T. cruzi* infection, treatment with serum from infected animals, *T. cruzi* lysate or with medium conditioned by the parasite (Figure 3). In *T. cruzi*-infected cultures, all compounds tested in a single dose pre-defined by cytotoxicity assays (benznidazole—100 µM, pirfenidone—1000 µM, losmapimod—30 µM and SP60015—10 µM) inhibited collagen stimulation, even though only benznidazole had a potent effect on infection rate, suggesting that the anti-fibrotic effect of pirfenidone, losmapimod and SP60015 is independent of infection reduction (Figure 3A). Remarkably, when the inflammatory approach was addressed by treating uninfected cultures with serum from infected animals, losmapimod presented a superior effect to benznidazole in inhibiting collagen stimulus in cardiac fibroblasts (Figure 3B). In the assays where uninfected cardiac fibroblasts were stimulated with lysate from *T. cruzi* trypomastigotes, only benznidazole and pirfenidone could inhibit the rise induced by collagen (Figure 3C). When the collagen stimulus of uninfected cells was performed with parasite-conditioned medium, only the inhibition of the c-Jun pathway through SP1600125 resulted in prevention of a rise in collagen in cardiac fibroblasts (Figure 3D).

**Figure 4:**
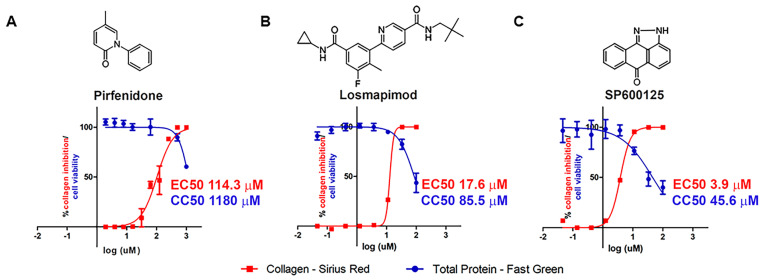
Dose-response effect of pirfenidone (**A**), losmapimod (**B**) and SP600125 (**C**) in collagen stimulation induced by *T. cruzi* in human cardiac fibroblasts. The Sirius Red/Fast Green assay for collagen detection allowed EC50 value calculation for collagen inhibition (Sirius Red readout, shown in red), while Fast Green allowed cytotoxicity assessment and calculation of CC50 for the host cell (Fast Green, shown in blue).

Our assay is sensitive enough to detect minor differences in collagen content and allows for the development of sigmoidal dose–response curves necessary for EC50 calculations. Pirfenidone treatment inhibited *T. cruzi*-induced collagen stimulation with an EC50 of 114.3 µM, which is high compared to other approaches, but falls inside the activity range and therapeutic window practiced in pulmonary fibrosis patients, who receive doses as high as 2000 mg/day with no severe adverse effects [26,45]. We could also build dose–response curves for losmapimod and SP600125, and we obtained an EC50 of 17.6 µM and 3.9 µM, respectively. Total protein detection through Fast Green allowed the determination of cytotoxicity using the same assay, with CC50 of 1170 µM for pirfenidone, 85.6 µM for losmapimod and 45.6 µM for SP1600125 (Figure 4).

**Figure 5:**
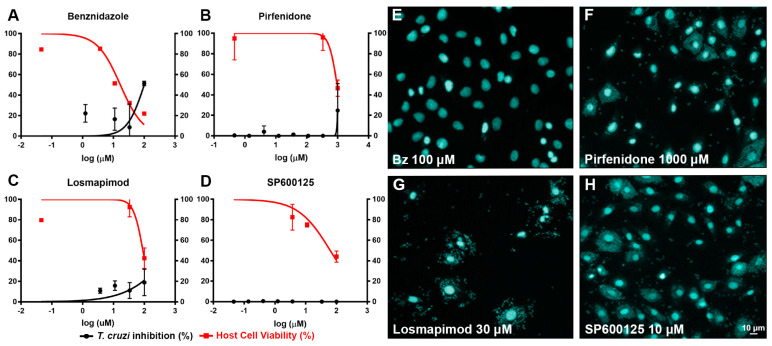
Trypanocidal activity in human cardiac fibroblasts infected with *T. cruzi*, treated with different compounds. Sigmoid dose-response curves for inhibition of *T. cruzi* infection revealed that only benznidazole (**A**,**E**) promoted a reduction in *T. cruzi* infection in human cardiac fibroblasts. Pirfenidone (**B**,**F**), losmapimod (**C**,**G**) and SP600125 (**D**,**H**) did not show the same efficacy in trypanocidal activity observed in human cardiac fibroblasts infected with *T. cruzi* (Brazil strain).

To investigate whether the anti-fibrotic activity of compounds was related to trypanocidal activity, cardiac fibroblast cultures infected with *T. cruzi* (Brazil strain) were treated with the studied inhibitors in concentration curves ranging from 0.04 to 100 µM. Image-based detection and quantification of *T. cruzi* infection showed that the treatments of human cardiac fibroblasts with pirfenidone (Figure 5B,F), losmapimod (Figure 5C,G) and SP600125 (Figure 5D,H) were not effective against *T. cruzi* infection. Only benznidazole (Figure 5A,E), the reference compound for Chagas disease treatment, presented a reduction in the percentage of infection in human cardiac fibroblasts. The image-based trypanocidal assay also allows the evaluation of cytotoxicity, and as the concentration of the tested inhibitors increases, cell viability is reduced to levels proportional to those obtained in assays with Sirius Red/Fast Green dye.

**Figure 6:**
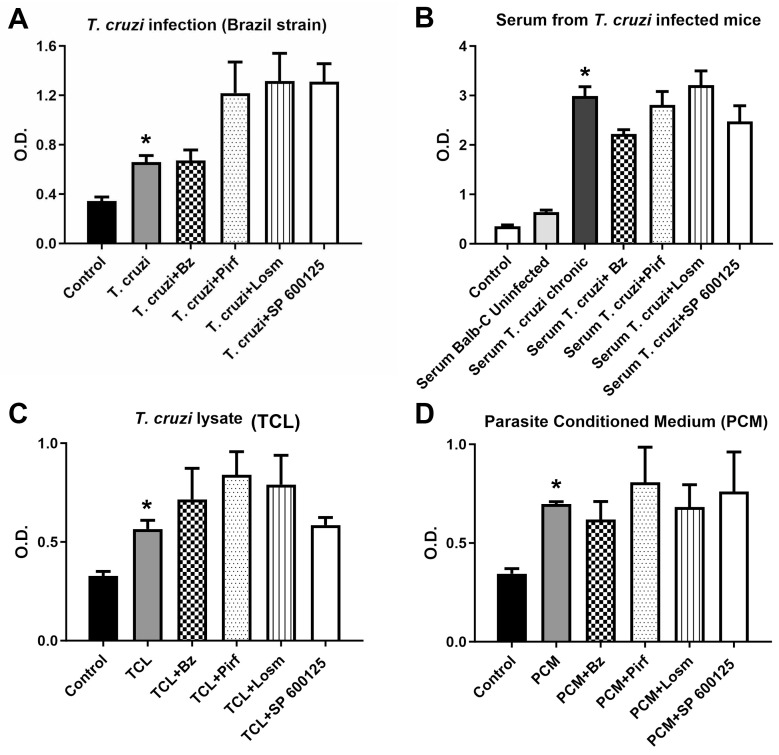
Proliferation of human cardiac fibroblasts evaluated by BRDU incorporation measured through ELISA. Proliferation of human cardiac fibroblasts infected with *T. cruzi* (**A**), stimulated with serum from *T. cruzi*-infected mice (**B**), *T. cruzi* lysate (**C**) or with parasite-conditioned medium (**D**) was analyzed by BRDU incorporation measured through ELISA. All conditions tested promoted an increase in human cardiac fibroblast proliferation. The treatment with benznidazole (100 μM), pirfenidone (1000 μM), losmapimod (30 μM) and SP600125 (10 μM) did not result in a reduction in human cardiac fibroblasts proliferation. * *p* ≤ 0.05 vs. control; unpaired Student’s *t*-test.

To continue to investigate the mechanisms by which the compounds were modulating collagen expression, we evaluated cardiac fibroblast proliferation by BRDU incorporation (Figure 6). We exposed human cardiac fibroblasts to the same conditions that resulted in collagen increase, and all conditions tested, i.e., direct *T. cruzi* infection, treatment with serum from infected animals, *T. cruzi* lysate or with medium conditioned by the parasite, also resulted in a significant increase in cellular proliferation (*p* ≤ 0.05 compared to uninfected/untreated controls). Treatment of the cardiac fibroblast cultures with pirfenidone, losmapimod or SP600125 did not affect cellular proliferation in any approach, with the differences in values being non-significant statistically (Figure 6).

### 2.2. In Vivo Assays

Considering the performance of the compounds in the in vitro assays, we moved forward to evaluate the potential of the signaling inhibitors as potential candidates to treat cardiac fibrosis in experimental models of *T. cruzi* infection in mice. We performed in vivo experimental assays using C57BL/6 mice infected with *T. cruzi*, Brazil strain, a host–parasite combination described to show hypertrophy and fibrosis during the chronic phase of the infection [46,47,48]. The animals were treated with benznidazole (100 mg/kg), pirfenidone (200 mg/kg), losmapimod (15 mg/kg) and SP600125 (3 mg/kg) at different stages of the disease. One group was treated at the late acute phase of Chagas disease at 60 days post infection (dpi), before fibrosis is established, and another group at 100 dpi, when the chronic phase of *T. cruzi* infection is more advanced, and symptoms are prominent.

**Figure 7:**
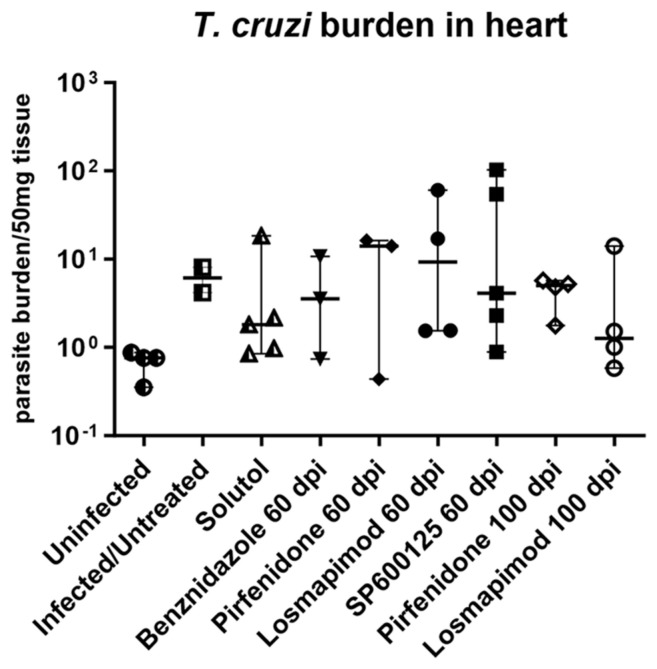
Parasitic load in cardiac tissue. Quantitative PCR revealed a parasitic load in the heart higher than the average plus 3 standard deviations of uninfected mice, showing infection, but at low levels. Treatment with signaling inhibitors such as pirfenidone, losmapimod and SP600125 did not modulate infection in cardiac tissue of C57BL/6 mice.

The presence of *T. cruzi* in the heart tissue was evaluated by quantitative PCR. All mice were positive for DNA for *T. cruzi*, despite the low levels of parasites detected. The signaling inhibitors pirfenidone, losmapimod and SP600125, administered to C57BL/6 mice infected with *T. cruzi* in different treatment regimens, did not result in a reduction in the parasitic load in cardiac tissue. These data are similar to the in vitro model after treatment with signaling inhibitors in cardiac fibroblasts infected with *T. cruzi*. (Figure 7).

**Figure 8:**
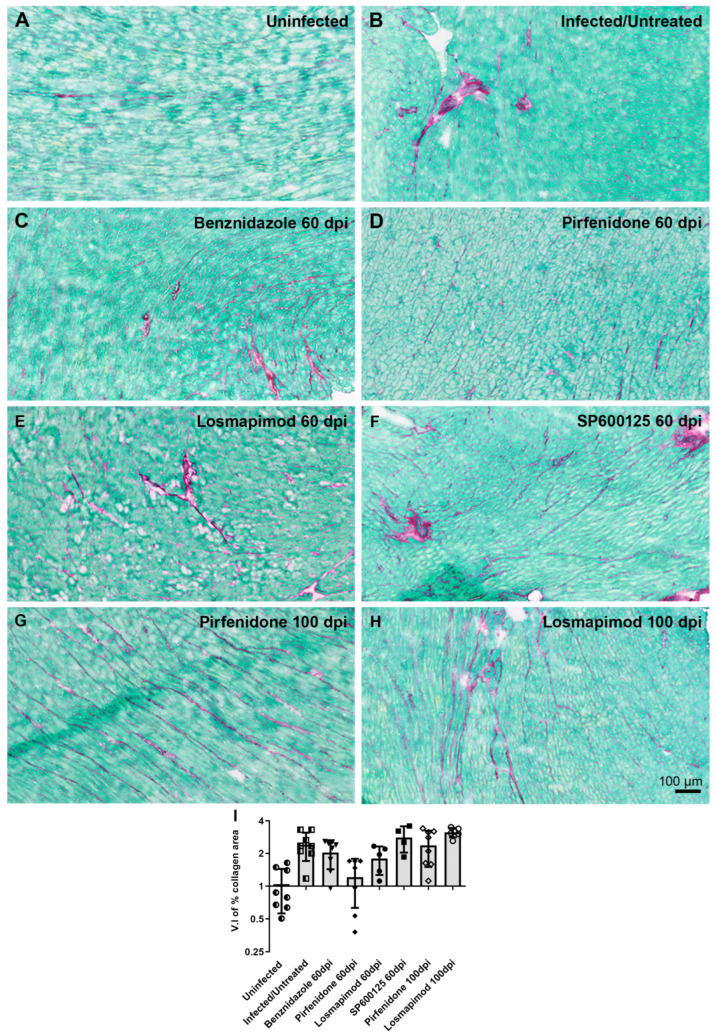
(**A**–**H**)Collagen analysis in cardiac tissue in C57BL/6 mice infected with *T. cruzi*. Heart tissue from C57BL/6 mice infected with *T. cruzi* (Brazil strain) was stained with Sirius red/Fast Green to label collagen (purple) and total proteins (green). Treatment with pirfenidone at 60 dpi reduced the total collagen expression in the ECM. The other inhibitors were not effective in reducing the total collagen after the treatment with 60 and 100 dpi. (**I**) Histological image analysis was performed using Image J software and the percentage of area occupied by total collagen staining is represented normalized as Variation Index (V.I.) when values are divided by the average of the values from uninfected animals (which are then considered = 1). The data showed that only pirfenidone at 60 dpi reduced total collagen in cardiac tissue. The other inhibitors did not show the same profile of the reduction in total collagen in the extracellular matrix in cardiac tissue infected with *T. cruzi*.

We analyzed the development of fibrosis in cardiac tissue in C57BL/6 mice infected with the *T. cruzi* Brazil strain, evaluating whether the compounds used in the study were effective in reducing collagen deposition in the heart’s matrix. Heart sections from mice treated after 60 and 100 dpi with benznidazole (100 mg/kg), pirfenidone (200 mg/kg), losmapimod (15 mg/kg) and SP600125 (3 mg/kg) were stained with Sirius Red/Fast Green. Untreated mice showed an increase in interstitial collagen deposits evidenced with Sirius Red (Figure 8B). After treatment with pirfenidone (200 mg/kg) at 60 dpi, a reduction in collagen expression in cardiac tissue was observed (Figure 8D). In contrast, treatment with benznidazole (100 mg/kg), losmapimod (15 mg/kg) and SP600125 (3 mg/kg) after 60 and 100 dpi were not effective in reducing collagen deposition in cardiac tissue (Figure 8B, Figure 8E and Figure 8F, respectively). Treatment with pirfenidone and losmapimod at 100 dpi did not result in a reduction in total collagen expression when compared with uninfected and untreated control tissue (Figure 8G,H). Image processing analysis with Image J software (https://imagej.net/ij/, accessed on 17 November 2023) revealed that the infected tissue showed a significant increase in collagen area when compared to control tissue. Treatment with benznidazole, losmapimod and SP600125 at 60 dpi was not effective in reducing the increase in collagen in cardiac tissue. In contrast, treatment with pirfenidone at 60 dpi promoted a reduction in the area occupied by collagen staining in the heart sections (Figure 8I).

**Figure 9:**
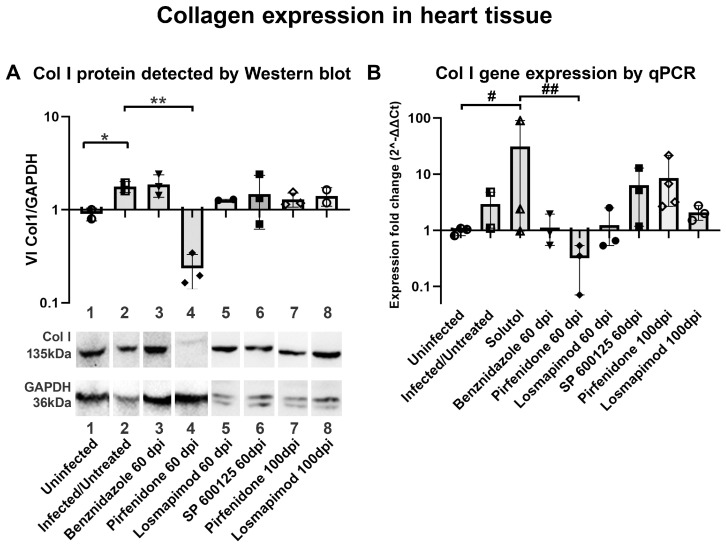
Collagen I expression in hearts of mice infected with *T. cruzi*. (**A**) Quantitative data by Western blot corroborated with qualitative data obtained by image processing analysis in which only pirfenidone prevented the increase in collagen in the ECM in treated cardiac tissue. (**B**) Measurement of the collagen I expression in the heart by quantitative PCR showed that only pirfenidone promoted an inhibition of collagen I gene expression after the treatment in the cardiac tissue infected with *T. cruzi*. * *p* ≤ 0.05 vs. uninfected (western blot); ** *p* ≤ 0.05 vs. infected/untreated; # *p* ≤ 0.05 vs. uninfected (qPCR); ## *p* ≤ 0.05 vs. Solutol.

Quantitative data obtained by Western blot (Figure 9A) and qPCR (Figure 9B) of heart tissue extracts from C57BL/6 mice infected with *T. cruzi* Brazil strain revealed that pirfenidone (60 dpi) was effective in preventing the increase in collagen type I triggered by *T. cruzi* infection, both at the gene and protein expression levels. However, treatment with other inhibitors, or pirfenidone at 100 dpi, did not show efficacy in reducing total collagen in the cardiac tissues of infected mice (Figure 9), confirming the measurements obtained from histological images for total collagen.

## 3. Discussion

Altogether, there are missing pieces in the puzzle of Chagas cardiomyopathy; on one hand, the reduction in parasitic load was not enough to resolve advanced chronic cardiomyopathy in patients [13]; on the other hand, studies show the correlation of fibrosis and reduced cardiac performance in Chagas cardiomyopathy [11], demonstrating the need to interfere with the fibrosis process to improve disease outcome. Currently, treatments provided to Chagas disease patients are trypanocidal compounds which do not have anti-fibrotic activities [49]. As there is no specific treatment for Chagas cardiomyopathy, the treatment used in these patients is symptomatic, with the use of diuretics, beta-blockers and angiotensin converting enzyme inhibitors [50]. Therefore, the identification of compounds that specifically inhibit and restore the cardiac tissue from Chagas disease fibrosis emerges as an alternative approach that needs to be prioritized.

Considering that previous data from our group showed that activation of SMADs, p38 MAPK and JNK signaling pathways occur concomitantly to the rise in fibronectin levels in the extracellular matrix of cardiac fibroblasts [21], we then evaluated the anti-fibrotic activity of the compounds pirfenidone, losmapimod and SP600125 that target these pathways. Pirfenidone is a synthetic inhibitor that has anti-inflammatory and anti-fibrotic properties in vitro and in vivo [51]. This inhibitor is used commercially in patients who develop idiopathic pulmonary fibrosis and acts on signaling pathways triggered by TNF-α and TGF-β, such as SMAD and MAPK signaling pathway proteins [52]. Losmapimod is a synthetic inhibitor of the p38 MAPK pathway, currently in clinical trials for facioscapulohumeral dystrophy [33,34]. SP600125 is a synthetic inhibitor of the JNK signaling pathway, with the potential to inhibit the existing isoforms JNK1, JNK2 and JNK3, with the JNK3 pathway being predominant in cardiac tissue [41].

Our study initially evaluated in which specific conditions a significant accumulation of extracellular matrix is detected in vitro. We analyzed the deposition of fibronectin and collagen in human cardiac fibroblasts stimulated with medium conditioned by *T. cruzi*, with parasitic lysate, with infected mice serum or directly infected by the pathogen. All conditions tested could induce a collagen increase in human cardiac fibroblasts. The treatment of uninfected cultures with serum from mice in the chronic phase of *T. cruzi* infection that presented cardiac fibrosis [53] mimics an inflammatory milieu and can contain pro-fibrogenic cytokines [54,55]. The collagen stimulation observed with the direct infection of cardiac fibroblasts by *T. cruzi* can also be explained by the induction of cytokine and NO secretion after the infection. There are reports that the primary culture of cardiomyocytes made up of a mixed population of cells (cardiomyocytes, cardiac fibroblasts and endothelial cells) produces chemokines, cytokines and nitric oxide independently of interaction with immune cells [56]. A spike on secretion of active TGF-β, the fibrosis landmark cytokine, is detected in the later stages of *T. cruzi* infection in similar multicellular heart-derived primary cultures [57]. Also, the parasite in the culture might cause secretion of parasitic factors, antigens and extracellular vesicles that can stimulate collagen in the cardiac fibroblasts. Other authors have shown that antigens secreted by the parasite, belonging to the family of trans-sialidases, resulted in an increase in the expression of fibronectin, collagen I and laminin in L929 fibroblasts [58], revealing the importance of these molecules secreted by the parasite in the synthesis and in the deposition of fibronectin and collagen in the extracellular matrix. Different proteomic approaches identified virulent factors such as trans-sialidases, mucins, mucin-associated surface protein (MASP), cruzipain and phosphatases in extracellular vesicles and secreted antigens of trypomastigotes [59,60]. These molecules have been implicated with parasitic evasion from the immune system and may act as immunomodulatory agents [59]. Parasitic antigens have been shown to promote the secretion of IL-10 and TNF-α by B cells, important cytokines in the immunomodulatory processes triggered by *T. cruzi* [61]. Antigens present in microvesicles secreted by the parasite, or even antigens remaining from *T. cruzi* lysis that remain in the cardiac interstitial space [62], can modulate the synthesis and deposit of extracellular matrix components in human cardiac fibroblasts, and are therefore important in the development of cardiac fibrosis in the chronic phase of Chagas disease.

The expression of extracellular matrix in cardiac fibroblasts can be influenced by cruzipain, a highly expressed parasitic protease, through the activation of latent TGF-β [63]. An increase in collagen in cardiac fibroblasts can be triggered by proteases in the lysate, activating this pro-fibrogenic cytokine. The inhibition of the increase in collagen by pirfenidone in cultures treated with this drug can also be explained by this mechanism, since pirfenidone inhibits TGF-β signaling. Pirfenidone has already been described as having an anti-fibrotic effect on cardiac fibroblasts [64]. Interestingly, when cardiac fibroblasts were stimulated with parasite-conditioned medium, only the inhibition of c-Jun by SP1600125 led to prevention of collagen accumulation. In vivo mouse models of dilated cardiomyopathy treated with SP600125 present a significant reduction in the expression of collagen and fibronectin, thus preventing the progression of cardiac fibrosis [65]. In reports of angiotensin II-induced fibrosis in atrial fibroblasts, the blockage of JNK signaling by SP1600125 resulted in reduced autophagy and an increase in collagen [66]. Imbalance in the autophagy process in the heart can cause cardiac fibrosis [67]. In the inflammatory approach, treating uninfected fibroblasts with serum from infected mice, the p38 MAPK inhibitor, losmapimod, presented a performance superior to the other compounds in preventing collagen accumulation. This inhibitor can interfere downstream with multiple cytokines’ signaling pathways, including TGF-β, TNF-α and IFN-δ signaling [68], preventing the stimulus of collagen by these inflammatory mediators. Other reports show that p38 inhibitors reduce total collagen production in ventricular fibroblasts through the myocardial transcription factor A (MRTF-A) [69]. Several lines of evidence have also shown that p38 MAPK is implicated with heart failure [70].

Human cardiac fibroblasts showed increased proliferation after treatment with medium conditioned with *T. cruzi*, with parasitic lysate, with infected mice serum or directly infected by the pathogen. All these different treatments/infections also resulted in collagen accumulation. Therefore, at least part of the increase in collagen expression with all different stimuli can be explained by enhanced cell proliferation. However, treatment of the stimulated cultures with the compounds did not affect the proliferation of cardiac fibroblasts. In contrast, other authors have shown pirfenidone was effective in inhibiting the proliferation of cultured rat cardiac fibroblasts but did not affect cell viability, suggesting that the anti-proliferative effect presented was not caused by a direct cytotoxic effect of pirfenidone [64]. The compounds also did not show any trypanocidal activity, and therefore, the inhibition of collagen accumulation can be credited to their specific mechanism of action and not to a potential reduction in parasitic load.

Since the compounds showed promising anti-fibrotic activity in different conditions in vitro, we moved forward to in vivo assays. We evaluated the parasitic load in cardiac tissue in *T. cruzi*-infected C57BL/6 mice. Our data showed that there was no significant difference in parasitic load in the heart by PCR after treatment with pirfenidone, losmapimod and SP600125. This information agrees with the in vitro model in which these compounds were not effective against *T. cruzi* infection in cardiac fibroblasts.

Our study revealed that only treatment with pirfenidone after 60 dpi was effective in preventing augmented collagen deposition in the hearts of *T. cruzi*-infected mice. Different compounds showed a similar anti-fibrotic effect. Early treatment of infected mice with verapamil promoted a reduction in inflammation and fibrosis in the acute phase of Chagas disease, while late treatment did not result in a beneficial effect [71]. Fibrosis in the heart is mediated at least partially by TGF-β, an important biomarker for the genesis of cardiac fibrosis in Chagas disease [16,72]. Additional inhibitors of the TGF-β pathway have been examined in experimental *T. cruzi* infection in vivo. The TGF-β receptor inhibitor GW788388 led to a reduction in parasitemia, mortality and prevented the development of fibrosis with a significant reduction in fibronectin and collagen I when administered to mice infected with *T. cruzi* in the acute phase [73]. The same compound was effective in reducing fibrosis and inflammation in models of chronic *T. cruzi* infection, and also resulting in improved cardiac function [74]. Neutralization of TGF-β by specific antibodies also resulted in an improvement in fibrosis and heart function during experimental chronic *T. cruzi* infection [75].

Pirfenidone is an oral drug used in the clinic to treat idiopathic pulmonary fibrosis with protective anti-fibrotic, anti-inflammatory and antioxidant activity in several models of cardiac fibrosis [27]. Different research groups reported that treatment with pirfenidone promoted an improvement in the cardiac fibrosis in mice with left ventricular hypertrophy induced by transverse aortic constriction (TAC), with inhibition of the TGF-β pathway and reduction in activated fibroblasts [30,76]. In mouse models of renal fibrosis, pirfenidone was effective in inhibiting extracellular matrix components associated with the epithelial-mesenchymal transition. The epithelial–mesenchymal transition gives rise to cardiac fibroblasts and is triggered by the p38 MAPK, c-Jun and ERK signaling pathways, which are inhibited after treatment with pirfenidone [77]. Pirfenidone also prevented intestinal fibrosis by regulating proliferation and apoptosis in interstitial fibroblasts, inhibiting the classic SMAD and PI3K/AKT signaling pathway [78]. To date, only one double-blind, randomized, placebo-controlled clinical trial to evaluate the effect of pirfenidone in preventing fibrosis in cardiac disease has been performed, the Pirfenidone in Patients with Heart Failure and Preserved Left Ventricular Ejection Fraction—PIROUETTE trial. Myocardial fibrosis, measured using magnetic resonance extracellular volume (ECV), was reduced by pirfenidone treatment over the course of 52 weeks in patients with preserved ejection fraction [79].

The prevention of fibrosis onset in the heart of our experimental chronic *T. cruzi* infection, together with promising results from the PIROUETTE clinical trial, suggest that pirfenidone is a strong candidate for repurposing as a combination treatment for Chagas disease. The drug is FDA-approved and is already on the market. Our proposal is to test the use of pirfenidone in combination with benznidazole, especially in indeterminate patients, to prevent the advancement of cardiac commitment through establishment of fibrosis, with potential beneficial outcome.

## 4. Materials and Methods

*Culture of cardiac fibroblasts*. Immortalized human cardiac fibroblasts were a kind gift from Dr. Tamer Mohamed, currently at the Baylor College of Medicine, Houston, TX, USA. They were generated from commercial primary human cardiac fibroblasts (Lonza, Basel, Switzerland) transfected with SV40 large T-antigen lentivirus for immortalization (Plasmid # 18922, https://www.addgene.org/18922 accessed on 17 November 2023; Addgene, Watertown, MA, USA). The cells were cultured in FBM Basal Medium (CC-3131; Lonza, Basel, Switzerland), supplemented with FGM-3 SingleQuot Supplements, containing 10% Fetal Bovine Serum, and insulin, hFGF, gentamicin and amphotericin in concentrations not disclosed by the manufacturer (CC-4525; Lonza, Basel, Switzerland). The cultures were kept at 37 °C in an atmosphere of 5% CO_2_. Expansion of human cardiac fibroblasts was performed with enzymatic dissociation of confluent cultures with trypsinization solution (0.0025% Trypsin, 0.01% EDTA in PBS). After trypsinization, cells were quantified in a Neubauer chamber and seeded at a density of 2 × 10^4^ cells/well in 96-well plates or in 24-well plates at a density of 5 × 10^4^ cells/well in FGM medium and kept at 37 °C in an atmosphere of 5% CO_2_.

*Parasites and infection of cultures*. Trypomastigotes of *T. cruzi* Brazil strain were kept through weekly passages in C2C12 mouse myoblast culture (CRL-1772; ATCC, Manassas, VA, USA), as described previously [80]. Human cardiac fibroblasts were infected at a multiplicity of 10 parasites/host cell.

*Preparation of parasite-conditioned medium (PCM)*. To obtain a medium conditioned by *T. cruzi*, we adapted the protocol of the collection of *T. cruzi*-released antigens as described previously [58]. Trypomastigote forms derived from cell culture (Brazil strain) were centrifuged for 5 min at 1000 rpm to remove cell debris. The supernatant was centrifuged again for 15 min at 3800 rpm and 2 × 10^8^ trypomastigotes were resuspended in 5 mL of FGM-3 containing 0.1% FBS. The parasitic suspension was maintained at 37 °C for 18 h. After that, the parasites were removed by centrifugation for 15 min at 3800 rpm and the supernatant was filtered through Millex 0.2 µM previously blocked with Fetal Calf Serum to guarantee absence of trypomastigotes. The microvesicles and proteins secreted by the parasites were concentrated on Centriprep 10 K (Millipore, Burlington, MA, USA) until it reached a final volume of 500 µL and stored at −20 °C. The protein content of the media was quantified through the Bradford assay. A total of 150 µg/well of proteins secreted by the parasite was used for the treatment of human cardiac fibroblasts.

*Obtention of T. cruzi lysate (TCL).* Media from infected C2C12 cultures having trypomastigotes from the *T. cruzi* Brazil strain released in the supernatant were centrifuged for 5 min at 1000 rpm to remove cell debris. The trypomastigote-containing supernatant underwent a second centrifugation at 3800 rpm for 15 min. Using a Neubauer chamber, the parasites were quantified. The pellet was then resuspended in 200 µL of PBS and subjected to 3 cycles of freeze–thawing, alternating between dry ice and a 37 °C water bath. The protein content was quantified using Bradford reagent. Then, 150 µg/well of proteins from *T. cruzi* lysate was used for the treatment of human cardiac fibroblasts.

*Collection of serum from control and T. cruzi-infected mice*. BALB-C female mice were routinely infected in the laboratory with *T. cruzi* CL strain holding a luciferase reporter gene for drug screening purposes, and the mice developed cardiomyopathy and fibrosis in the chronic phase of infection [53]. Trying to minimize the number of animals used in research, we kept the infected and untreated mice from drug screening assays until 120–180 days after infection, and after euthanasia, total blood was collected through cardiac puncture from each animal into tubes containing gel-clotting activator. After centrifugation, the serum was stored at −20 °C and later added to human cardiac fibroblast cultures at 20% in FGM-3. All procedures involving the handling of animals were approved by the Ethics Committee of the UCSD (Institutional Animal Care and Use Committee, IACUC) under protocol number S14187.

*Collagen measurement*. Human cardiac fibroblasts stimulated with *T. cruzi* lysate or PCM or serum of infected mice, or directly infected by *T. cruzi* Brazil strain (48 h of infection), were initially treated with 100 µM benznidazole (Sigma Chemical Co., St. Louis, MO, USA), 1000 µM pirfenidone (Selleckchem, Houston, TX, USA), 30 µM Losmapimod (Selleckchem, Houston, TX, USA) and 10 µM SP60015 (Selleckchem, Houston, TX, USA). For the direct infection model, concentration curves ranging from 0.04 to 100 µM were also performed for benznidazole, losmapimod and SP600125. Pirfenidone was added in curves at 0.45–1000 µM. After 72 h of treatment, the cultures were fixed in Kahle solution (4% formaldehyde, 30% ethanol and 2% acetic acid) and stained with Sirius Red/Fast Green dye (0.1% Sirius Red/0.1% Fast Green in saturated picric acid solution) for 2 h, which allows the semi-quantitative measurement of collagen content and non-collagen proteins in culture [81]. After washing with distilled water, the dye was extracted from the cells with an extraction buffer (0.1 N NaOH/Methanol 1:1). The supernatant was transferred to a new plate, and the extracted dyes were read in microplate reader EnVision Multilabel Plate Reader (Perkin Elmer, Waltham, MA, USA) at λ 540 and 605 nm. The amount of collagen in each sample was calculated after corrections due to absorbance shoulders between the two dyes, following the formulas: Collagen (µg/sample) = OD 540 value − (OD 605 value × 0.291)/0.0378; non-collagenous proteins (µg/sample) = OD 605 value/0.00204. Variations in collagen (Δ collagen) were calculated subtracting uninfected control sample values (µg) from *T. cruzi*-infected sample collagen values (µg). Inhibition of rise in collagen was calculated following the formula: Inhibition = 100 − (Δ collagen compound treated sample × 100/mean of Δ collagen untreated and infected controls).

*Indirect Immunofluorescence.* Human cardiac fibroblasts were seeded in 24-well plates with round glass coverslips coated with gelatin 0.1% for 20 min at 4 °C and treated with PCM for 72 h. The cells were fixed for 5 min at room temperature with 4% paraformaldehyde (PFA) in PBS followed by washing in PBS. To block nonspecific reactions, the fixed cultures were washed (3 × 20 min) with PBS containing 4% bovine serum albumin (BSA). The coverslips were then incubated for 18 h at 4 °C with anti-fibronectin antibody (1:400; Sigma Chemical Co., St. Louis, MO, USA). After successive washes in PBS, the cultures were incubated for 1 h at 37 °C with secondary anti-rabbit antibody conjugated with Alexa 546 (1:1000; ThermoFisher Scientific, Waltham, MA, USA). For visualization of the nucleus, cells were stained with 4′, 6-diamidino-2-phenylndole (DAPI–DNA dye); then, the coverslips were mounted in Vectashield Antifade Mounting Medium (Vector Laboratories, Newark, CA, USA) and sealed with nail polish. The images were acquired at the Microscopy Core of UCSD using an Olympus confocal laser scanning microscope.

*Evaluation of trypanocidal activity.* To evaluate the effect of signaling pathway inhibitors on *T. cruzi* infection, human cardiac fibroblasts were cultured in supplemented FGM-3 Fibroblast Growth Medium-3, as described above. Human cardiac fibroblasts were plated at a density of 1.5 × 10^4^ cells/well in 96-well flat-bottom, µclear black plates (# 655090; Greiner Bio-One, Kremsmünster, Austria), and infected with trypomastigotes of *T. cruzi* Brazil strain at 10 parasites:1 host cell ratio, in a volume of 50 µL. Immediately after infection, the cultures were treated with benznidazole, pirfenidone, losmapimod and SP600125 by adding 50 µL of media containing 2× the final concentration of the compounds. Concentration curves ranging from 0.04 to 100 µM were performed for benznidazole, losmapimod and SP600125. Pirfenidone was added in curves at 0.45–1000 µM. The culture was incubated at 37 °C and 5% CO_2_ atmosphere for 72 h of infection and treatment of the compounds. The cells were then fixed with 4% paraformaldehyde for at least 1 h and stained with 0.5 10 μg/mL of 4′, 6-diamidino-2-phenylndole (DAPI–DNA dye) for 4 h. The plates were then photographed at 10× with ImageXpress Micro XL (Molecular Devices, San Jose, CA, USA), 4 fields per well. The images were analyzed using MetaXpress software (https://www.moleculardevices.com/products/cellular-imaging-systems/acquisition-and-analysis-software/metaxpress, accessed on 17 November 2023) with algorithms that detect and quantify the host cell nucleus and the parasite’s kinetoplast by circularity and size (125 μm^2^ for host nucleus, and 1–2 μm^2^ for parasite nucleus/kinetoplast). Antiparasitic activity was normalized based on negative controls (infected wells having only DMSO) and positive controls (uninfected wells). The number of host cells was also used to quantify the cytotoxicity of the compounds.

*Proliferation measurement.* Proliferation of *T. cruzi*-infected human cardiac fibroblasts treated with signaling inhibitor compounds was measured with BrdU Cell Proliferation ELISA Kit (Millipore, Burlington, MA, USA), following the manufacturer’s instructions. For the assay, cells were trypsinized and plated at a density of 1.5 × 10^4^ cells/well in 96-well, flat-bottom plates. Human cardiac fibroblasts were infected with *T. cruzi* Brazil strain or stimulated with *T. cruzi*-conditioned medium (150 µg/mL), parasitic lysate (150 µg/mL) or serum from uninfected and *T. cruzi*-infected mice (20%). Cultures were treated for 72 h with 100 µM benznidazole (Sigma Chemical Co., St. Louis, MO, USA), 1000 µM pirfenidone (Selleckchem, Houston, TX, USA), 30 µM Losmapimod (Selleckchem, Houston, TX, USA) and 10 µM SP600125 (Selleckchem, Houston, TX, USA). After treatment, cells were incubated with 5-Bromo-2′-deoxyuridine (BrdU), a synthetic thymidine analogue, for 2 h. The cells were then fixed for 30 min, and the BrdU particles incorporated by proliferating cells were revealed by the colorimetric detection of the peroxidase activity on the antibody–antigen complex. Data were collected in the microplate reader EnVision Multilabel Plate Reader (Perkin Elmer, Waltham, MA, USA) at λ 450 nm.

*Animals.* C57/Bl6 male mice, six weeks old, were obtained from Jackson Laboratories (Bar Harbor, ME, USA). These mice were housed in groups of up to five animals per cage within a standard room kept at a temperature range of 20 to 24 °C, following a 12-h light and 12-h dark cycle. The mice had unrestricted access to sterilized water and standard chow. All animal-related procedures were conducted following the guidelines of the Institutional Animal Care and Use Committee at the University of California San Diego, under protocol number 14187, overseen by Jair L Siqueira-Neto.

*T. cruzi infection of mice.* The Brazil strain of *T. cruzi* was selected for investigation during the chronic phase because of multiple reports showing the survival of mice during the acute infection stage, with evidence of pathophysiological changes in the heart during the chronic stage of infection, including chronic inflammation, fibrosis and hypertrophy [46,47,48]. C57/Bl6 male mice were infected with *T. cruzi* Brazil strain with an inoculum of 1 × 10^4^ trypomastigotes/animal via the intraperitoneal route. Then, 20 days post infection (dpi), screening for the presence of parasites in the blood was conducted. A 5 µL drop of blood was collected from the tail vein of each animal, and *T. cruzi* infection was confirmed if trypomastigotes were visualized through microscopy. Only animals with positive parasitemia were used in the study.

*Treatment Protocols*. Two treatment regimens were performed. Treatment started at the late acute phase, 60 dpi, before the establishment of fibrosis, or at 100 dpi, when fibrosis was already installed. The animals were treated for 28 days with pirfenidone (200 mg/kg, Selleckchem, Houston, TX, USA), an inhibitor of the TGF-β signaling pathway; losmapimod (15 mg/kg, Selleckchem, Houston, TX, USA), p38 MAPK inhibitor; SP600125 (3 mg/kg, Selleckchem, Houston, TX, USA), JNK inhibitor; and benznidazole (100 mg/kg; Sigma, St. Louis, MO, USA) as control. The compounds were diluted in 10% Solutol (Kolliphor; Sigma, St. Louis, MO, USA) and administered once a day orally via gavage (pirfenidone, losmapimod and benznidazole) or intraperitoneally (SP600125). In both approaches, general health was checked during treatment. After the end of the treatment period, the animals were euthanized in a CO_2_ chamber, and the hearts were collected for evaluation of parasitic load by PCR, histological processing for ECM analysis by Sirius Red/Fast Green staining and collagen measurement by Western blot and quantitative PCR.

*Quantitative PCR*. At the end of the treatment period, mice were euthanized and quickly perfused with PBS. Their hearts were removed, cleaned and briefly washed in PBS. A small apical section of each heart was weighed and preserved in RNA-later. At least 50 mg of tissue was homogenized using a ZR BashingBead Lysis Tube (2.0 mm; Zymo Research, Irvine, CA, USA) and DNA and RNA were purified simultaneously using a Quick-DNA/RNA™ Miniprep Plus Kit (Zymo Research, Irvine, CA, USA). Quantitative PCR (qPCR) for *T. cruzi* detection was performed as described previously [82,83]. Briefly, 180 ng of DNA was used as a template for qPCR using Lightcycler 480 Sybr green I Master mix (Roche, Basel, Switzerland) on a Stratagene Mx3005P RT-PCR thermocycler. The parasite satellite DNA region was detected with primers ATCGGCTGATCGTTTTCGA and AATTCCTCCAAGCAGCGGATA, and mouse TNF-α was detected with primers TCCCTCTCATCAGTTCTATGGCCCA and CAGCAAGCATCTATGCACTTAGACCCC. The thermal profile was composed of 95 °C for 10 min, followed by 40 cycles of 95 °C for 30 s, 58 °C for 60 s, and 72 °C for 60 s. To determine the parasitic burden in 50 mg of tissue, a standard curve was established with uninfected mice heart samples spiked with 2 × 10^7^ *T. cruzi* epimastigotes. The standard curve was generated through serial 10-fold dilutions in DNA from uninfected mice, resulting in a curve ranging from 2 to 200,000 parasite equivalents. *T. cruzi* satellite DNA values were normalized with mouse TNF-α detection, and the delta Ct from these two genes was calculated for all samples. The standard curve was performed at each measurement, and the parasitic load for each sample was calculated from the equation of the linear regression of the curve. Mice were positive for *T. cruzi* infection if the parasitic burden was higher than the average plus 3 standard deviations of uninfected mice. To evaluate collagen I gene expression, c-DNA was generated from RNA with SuperScript™ IV First-Strand Synthesis System (ThermoFisher, Waltham, MA, USA). Then, 4000 ng of c-DNA was used as template for qPCR using Lightcycler 480 Sybr green I Master mix (Roche, Basel, Switzerland) on a Stratagene Mx3005P RT-PCR thermocycler. Collagen I gene expression was quantified with primers CCTGGTAAAGATGGTGCC and CACCAGGTTCACCTTTCGCACC, normalized with GAPDH through primers GACTTCAACAGCAACTCCCAC and TCCACCACCCTGTTGCTGTA [84,85]. The thermal profile comprised 1 min at 95 °C and 40 cycles of 95 °C for 30 s, 57 °C for 30 s, and 72 °C for 30 s. Collagen expression values were normalized with mouse GAPDH detection, and the expression fold change calculated with 2^−ΔΔCt^ for all samples.

*Western blot*. Half of the hearts of C57Bl/6 mice infected by *T. cruzi* Brazil strain at 60 and 100 dpi, treated with benznidazole, pirfenidone, losmapimod and SP600125, were fragmented and mechanically macerated with a pestle and mortar using 500 µL of lysis buffer (50 mM Tris, 150 mM NaCl, 1% Triton X-100) and protease inhibitor cocktail (Sigma Chemical Co., St. Louis, MO, USA; AEBSF–[4-(2-Aminoethyl) benzenesulfonyl fluoride-hydrochloride]; aprotinin, bestatin hydrochloride, E-64–[N-(trans-Epoxysuccinyl)-L-leucine 4-guanidinobutylamide]; Leupeptin hemisulfate salt, Pepstatin A). Aliquots of 20 µL were previously separated from each sample for protein measurement using the Folin–Lowry method. To the remaining extracts, 5× sample buffer (0.3 M Tris, 10% SDS, 0.125% Bromophenol Blue, 25% β-mercaptoethanol, 50% Glycerol) was added, and the samples were boiled in a dry bath at 100 °C for 5 min immediately after extraction, for complete inactivation of proteases and phosphatases. All extracts were stored at −20 °C. After protein measurement, total proteins obtained from heart tissue extracts were subjected to polyacrylamide gel electrophoresis using 10 µg of protein in polyacrylamide gel 10% for the detection of Collagen I. The separated proteins were transferred to a nitrocellulose membrane and incubated with blocking buffer (Tris Buffered Saline, 5% non-fat milk, 0.1% Tween 20) for 1 h at room temperature. After blocking, the membranes were incubated with anti-Col1A1 antibody (1:2500; ABclonal Technology, Woburn, MA, USA), incubated for 18 h at 4 °C. The membranes were then washed and then incubated for 1 h at room temperature with an anti-GAPDH antibody (1:100; Santa Cruz Biotechnology, Dallas, TX, USA) as an internal control. The reaction was revealed by peroxidase-conjugated anti-rabbit and anti-mouse secondary antibodies (Pierce Biotechnology, Waltham, MA, USA), both diluted 1:10,000 in blocking buffer. Then, the membranes were washed with TBS + 0.1% Tween-20, and peroxidase was revealed by chemiluminescence, using the Super Signal West Pico kit (Pierce Biotechnology, Waltham, MA, USA). Band images were obtained digitally with ChemiDoc MP Imaging System (Bio-Rad, Hercules, CA, USA). Densitometry of the resulting bands was performed using the Image J program (https://imagej.net/ij/, accessed on 17 November 2023). Densitometry values were normalized, and the variation index (V.I.) was calculated considering the uninfected control = 1.

*Histology and histopathology analysis.* Upon euthanasia, the heart was halved sagittally, embedded in Tissue-Tek O.C.T., and snap-frozen in liquid nitrogen. Samples were sectioned in a cryostat, fixed in buffered formalin and stained with Sirius Red/Fast Green. The slides were scanned using a Nanozoomer Slide Scanner (Hamamatsu Photonics, Hamamatsu City, Japan) and images were obtained through NDP viewer software (https://www.hamamatsu.com/jp/en/product/life-science-and-medical-systems/digital-slide-scanner/U12388-01.html, accessed on 17 November 2023) (Hamamatsu Photonics, Hamamatsu City, Japan). To quantify fibrosis levels, five random images of cardiac tissue (10× magnification) were extracted from each scanned heart section. The fibrosis area was segmented by color using FIJI software (https://imagej.net/software/fiji/, accessed on 17 November 2023) [86], and the area occupied by Sirius Red staining was quantified.

*Statistical analysis*. In vitro assays were analyzed with unpaired Student’s *t*-tests. Data from in vivo experiments were analyzed with non-parametric tests by Mann–Whitney and/or Kruskal–Wallis. Differences were considered statistically significant when *p* ≤ 0.05.

## Data Availability

Data is contained within the article.
